# The Relation Between Human Values and Perceived Situation Characteristics in Everyday Life

**DOI:** 10.3389/fpsyg.2018.01676

**Published:** 2018-09-13

**Authors:** Rebekka Kesberg, Johannes Keller

**Affiliations:** Department of Social Psychology, Ulm University, Ulm, Germany

**Keywords:** human values, situation perception, 8 DIAMONDS, day reconstruction method, daily life

## Abstract

Values refer to abstract beliefs which serve as guidelines in peoples’ life and affect the way people and events are evaluated. Simultaneously, unlike attitudes, values transcend specific actions, and situations. While recent research showed that values are related to the attention and interpretation of situational information in standardized laboratory settings, up to date hardly any empirical work investigated how values relate to situation perception in daily life. In our study, we assessed the relation between the endorsement of human values and situation characteristics (i.e., the 8 DIAMONDS). Using the Day Reconstruction Method in two samples (German and US-American), we found that especially variance in the experience of negatively connoted situation characteristics were due to individual differences. Power was related to experiencing more deceptive situations, while the reversed pattern emerged for universalism and benevolence. Tradition was related to experiencing more aversive situations while self-direction was related to experiencing less situations high in adversity. Although, our results might provide some initial evidence for a relation between personal values and subjective situations experiences in everyday life, no clear pattern emerged and further investigation of the relation is necessary.

## Introduction

In his famous formula B = *f* (P, E), Lewin (1939) proposed that behavior (B) is a function of the person (P) and the environment (E). More precisely, behavior is a function of a person’s characteristics and his or her subjective experiences of the environment, but not necessarily determined by objective aspects of the environment. While there has been abundant research on how personality traits or differences in physical environment relate to behavior, up to date, subjective situation experience has mostly been overlooked (Rauthmann et al., 2014). One major reason could be that while there are numerus and widely accepted taxonomies (e.g., the Big Five) to capture individual differences in personality traits; a generalized and accepted taxonomy to capture individual differences in situation experiences has been missing. However, in recent years the investigation of differences in the subjective situation experiences has become more and more popular, and thus various instruments to measure so-called situation characteristics have been published (see the overview by Horstmann et al., 2017). This new development enables us to obtain a more precise and comprehensive picture of human behavior as a function of individual differences, like personality traits or motivational orientations, and subjective situation experiences. The current work attempts to provide evidence on how basic motivational orientations (i.e., the Schwartz model of basic human values) relate to subjective situation experiences (i.e., the situational 8 DIAMONDS), and to behavior in everyday life. Using the Day Reconstruction Method (DRM; Kahneman et al., 2004) allows us to obtain information about peoples’ activities and contacts in everyday life, and how they subjectively experienced these situations. Our study advances previous research on how values relate to situational factors by using a novel approach to measure psychological relevant aspects of situations. In the following section we will introduce the concepts and discuss theoretical assumptions about the relation between values and subjective situation experience.

Values are abstract and context-independent beliefs about what people want to achieve in life, e.g., power. Values are motivational goals which refer to desirable end-states (Schwartz, 1992). There are numerous values and each person holds a variety of values at the same time which differ in their importance (Schwartz, 1992; Bardi and Schwartz, 2003). Over the decades, many different constructs and theories evolved around values (e.g., the equality-freedom model of ideology proposed by Rokeach, 1973). Up to date the most prominent one is the model of Basic Values proposed by Schwartz (1992). The model assumptions have been extensively studied within different samples and in over 70 countries (Schwartz and Rubel, 2005). Schwartz proposed 10 basic human values which differ in their underlying motivational base: benevolence, universalism, conformity, security, tradition, power, achievement, hedonism, stimulation, and self-direction.

One key feature of the model are the detailed assumptions about the interrelation between values, i.e., compatibility and conflict between values. These conflicts and compatibilities between values can be modeled in a circular structure representing a motivational continuum. In this circumplex model, values which are adjunctive represent compatible motivational orientations while those on the other end of the circle represent opposing motivational orientations. The further away two values are located within the circle, the more dissimilar are their underlying motivations (Schwartz, 1992). Within this motivational continuum, the 10 originally proposed values can further be divided into 19 more narrowly defined values (Schwartz et al., 2012) or grouped into four higher order constructs based on two major dimensions.

The first dimension is the *self-enhancement self-transcendence dimension* (SET). Self-enhancement mainly consists of the values achievement and power as well as some part of hedonism. People valuing self-enhancement believe that for example success as well as showing competence is important in life. On the other side of that pol are self-transcendence values, namely benevolence and universalism. People who value self-transcendence believe that, e.g., equality and caring for others is important.

The second dimension is the *openness-to-change conservation dimension* (OC). The values self-direction and stimulation form the openness-to-change dimension; therefore, people valuing openness-to-change attribute high importance to creativity, freedom, and self-determination. On the other side of this pol is the conservation dimension consisting of conformity, security, and tradition. People valuing conservation believe it is important to, e.g., maintain the status quo and live in a safe surrounding. While the two pols of each dimension refer to opposing values, the dimension themselves are conceptualized as independent, e.g., a person valuing self-transcendence does not necessarily value openness-to-change.

Furthermore, there are some central assumptions about values. Among other things, they are supposed to transcend specific actions and situations, and at the same time they are standards which are used to evaluate people and events (Schwartz, 1992). The first assumption implies that values represent motivational goals which are of importance independent from the specific task or situational factors. For example, a person who is concerned with protecting nature (part of universalism value) should try to act environment-friendly (at least to some extent) at home, but also in public or at the workplace. The second assumption implies that values are used to judge situations and their opportunities as well as consequences based on individual values. In combination, these assumptions suggest that situations people encounter in everyday life are overall judged based on individual values, and independently of specific factors, all encountered situations should be judged using the same value. Previous research has investigated how values relate to situational aspects (e.g., in a cooperative framed decision task valuing self-transcendence was related to cooperative behavior, Sagiv et al., 2011). However, none of those studies has measured *perceived subjective situational differences* using a valid and standardized instrument. Fortunately, recently a taxonomy has been developed which enables us to measure subjective differences and to capture psychologically relevant aspects of a situation, as outlined in what follows.

Research investigating situational factors has often focused on the situation as a whole, e.g., framing of situations (Tversky and Kahneman, 1981), saliency of stimuli (e.g., Wit and Kerr, 2002), interpersonal communication (e.g., Tazelaar et al., 2004) and group size (e.g., Brewer and Kramer, 1986; De Cremer and Leonardelli, 2003). For example, studies investigating the bystander effect manipulated objective differences (i.e., number of people present) to examine differences in behavior. While the results show differences in behavior depending on the manipulation, i.e., depending on objective differences in the situation, the results do not allow drawing conclusions about differences in the subjective experience of the situation. Depending on the research question, examining subjective situation experience might sometimes not be relevant. However, to fully assess and understand how people act and feel, measurement of the situations they subjectively experience is needed (Benet-Martínez et al., 2015).

In the last decades, many situation taxonomies were developed to measure situations. Up to date, however, none has found widespread acceptance (e.g., Moos, 1973; Van Heck et al., 1994; Kelly, 2003). Therefore, contrary to the assessment of personality traits, there still is no consensus on how to define and assess situations (e.g., Hogan, 2009; Rauthmann et al., 2015a). Most approaches build on the theoretical background that any given situation can be described using three aspects, namely *cues, characteristics*, and *classes*. *Cues* refer to physical stimuli which can be objectively quantified in a situation, e.g., how many people are present or which objects are present. Generally, people should agree about situation cues, e.g., either there is a table in the room or there is no table in the room. Cues are the most frequently inquired aspects of situations in psychological studies (e.g., there are other people present or not). *Characteristics* are used to describe psychological relevant aspects of situations (e.g., a fearsome situation). Situation characteristic should not be mistaken for the overall affective ratings of situation by the person in the situation. For example, people may agree that a situation is negative, but the specific affective reaction could be anxiety, anger or sadness. *Classes* refer to groups of situations which are clustered together based on similar cues or characteristics, which are used to describe these situations. For example, “meeting friends” and “teaching a class” could both be grouped into the class “social situation,” although there are differences in cues and characteristics.

From a psychological perspective, situation characteristics might be the most interesting aspects of situations as they measure the psychological (subjective) meaning of perceived situational cues (Rauthmann et al., 2014). Hence, they may be better predictors for behavior than objective situational cues. Sherman et al. (2013) showed that situations with similar characteristics evoke similar behavior independent of the situation cues. Recently, Rauthmann et al. (2014) proposed that situation characteristics can be captured in a parsimonious taxonomy, which can be used to classify and compare situations. Based on one frequently used measure for situational characteristics (i.e., Riverside Situational Q-Sort; Wagerman and Funder, 2009), they identified eight major situation characteristics: The situational 8 DIAMONDS. Those dimensions are: Duty, Intellect, Adversity, Mating, pOsitivity, Negativity, Deception, and Sociality. Duty captures to what extent a situation is perceived as containing work, attending to tasks, making decisions and fulfilling duties. Intellect captures to what extent a situation is perceived as containing intellectual and cognitive demands as well as possibilities to show intellectual prowess. Adversity captures to what extent a situation is perceived as containing problems, threats, conflicts, and criticism. Mating captures to what extent a situation is perceived as containing opportunities for sex, love and romance, that is finding or maintaining potential mates. Positivity captures to what extent a situation is perceived as pleasant, easy, clear, and enjoyable. Negativity captures to what extent a situation is perceived as containing the possibilities for any kind of negative feelings (e.g., frustration, anger, etc.) to emerge. Deception captures to what extent a situation is perceived as containing opportunities for betrayal, deception and hostility. Sociality captures to what extent a situation is perceived as containing possibilities for socializing, relationship formation, and interpersonal warmth. The *Situational Eight* emerged as dimensions on which different raters (*in situ* and *ex situ*) substantially agreed showing that even if people themselves did not actually experienced a situation they agreed on how to characterize the situation along the dimensions (Rauthmann et al., 2014).

The 8 DIAMONDS are also related to situational cues, e.g., working was characterized by high duty and negativity as well as low positivity (Rauthmann et al., 2014). In addition, the 8 DIAMONDS are associated with a wide range of self-rated behavior, e.g., behaving competitive was positively related to the experience of deception and negatively to sociality (Rauthmann et al., 2014). Despite their short history, the 8 DIAMONDS have been widely used. For example, Brown and Rauthmann (2016) investigated the relation between age and situation characteristics showing that mean-level patterns are related to opportunities and constraints at various ages (e.g., duty peaked among those people in their 40s which can be considered a phase in which working and caring for a family is common). Serfass and Sherman (2015) collected and rated situation information given in Twitter tweets over a period of 2 weeks. They found that during typical working hours (9 a.m. to 5 p.m.) tweet information described situations high in duty, while sociality peaked in the late afternoon and early evening. Overall, the tweets describing deceptive or aversive situations were low. In line with their finding, Guillaume et al. (2016) compared situation experiences across cultures finding that on average people around the world experience similar and largely pleasant situations in the evening. The usefulness of considering situation characteristics to investigate human experiences was also shown in a study by Kocjan and Avsec (2017). Their results indicate that situations high in positivity and intellect promote flow experiences. Using the Experience Sampling Method (ESM), Sherman et al. (2015) found that the 8 DIAMONDS predicted behavior independent of personality. Their results showed that for example people who on average reported experiencing more deception showed less honest behavior.

Recent research has shown that people mostly agree on the characteristics of situations (Rauthmann et al., 2014), and that on average 70% of the variance in situation experiences is due to differences between situations. However, that implies that 30% of the variance is due to individual differences (Sherman et al., 2015), and it has been shown that situations experienced over time by one individual tend to be more similar to each other compared to situations experienced by others (Sherman et al., 2015). For example, people who scored high on extraversion, reported to experience more situations high in sociality (Sherman et al., 2015). Another study found that distinctiveness of situation stimuli construction is associated with personality (Todd and Funder, 2012). Taken together these studies show that people, at least partly, shape their experienced situations and therefore their behavior may be based on a subjective experience of situations.

In order to understand individual differences in situation perception, recent research has mainly focused on personality traits (Rauthmann et al., 2014). However, situation characteristics often capture perceived opportunities and requirements for the emergence of different emotional, cognitive and behavioral outcomes in situations. Studies have shown that values lead to giving more attention to information cues that are consistent with one’s personal values or risk the attainment of those values (Crick and Dodge, 1994; Verplanken and Holland, 2002). Based on those findings, it seems plausible that values are also related not just to cues, but also to perceived situation characteristics, especially considering that Schwartz (1992) proposed that values are used to evaluate actions, policies, and people.

There are two main ways how values could refer to situation experience: (1) by situation selection and (2) by situation construal (Rauthmann et al., 2015b). Situation selection means that people consciously or unconsciously seek out situations which fit for example to their values. Situation construal refers to the distinct subjective interpretations of situational cues due to individual differences. Up to date, there has been no empirical investigation of the relation between values and situation selection or situation construal. The present contribution addresses this gap in research by examining how values relate to situation characteristics in everyday life.

The relation between Schwartz values and the Big Five (Roccas et al., 2002; Fischer and Boer, 2015; Parks-Leduc et al., 2015) as well as the relation between the Big Five and situation characteristics (Rauthmann et al., 2014, 2015b) have been examined. Extraversion was positively related to experiencing sociality and adversity as well as positively to self-direction, stimulation, hedonism, achievement and power. The conservation values – security, conformity and tradition – were negatively related to extraversion. Openness was positively related to intellect and universalism, self-direction and stimulation; while it was negatively related to conservation values and power. Agreeableness was positively related to sociality and to self-transcendence values, conformity and tradition. Agreeableness was negatively related to adversity and deception, as well as openness-to-change values and self-enhancement values. Conscientiousness was positively related to duty and sociality, and achievement as well as security and conformity. It was negatively related to adversity, negativity and deception, as well as to universalism and stimulation. Neuroticism was positively related to negativity and tradition, and negatively to positivity and achievement.

Based on those and other findings as well as theoretical considerations (i.e., the circumplex structure of the value model), we make several specific assumptions about the relation between values and situation characteristics.

### Duty

We assume that conformity, tradition, security as well as achievement are positively related to Duty. On a conceptual level, it seems plausible that especially conformity has a strong relation with duty. Conformity entails the tendency to comply with rules and the avoidance of harming social norms. Therefore, we assume that people valuing conformity are more likely to experience situations high in Duty. Additionally, achievement, but not power should be positively related to duty. Experiencing duty refers to, e.g., task-orientated thinking and focusing on minor details, which on a conceptual level seems closer to valuing achievement (i.e., showing competence, being ambitious) compared to power (i.e., authority, social prestige). There are different ways to fulfill achievement values, and paying attention to details or working carefully, may be one way to show competence. Moreover, studies have shown that people valuing achievement are willing to study late at night although they are already well-prepared for an exam. This behavior might also be a part of experiencing a sense of duty to study.

### Intellect

Like the personality trait openness, we assume that universalism as well as stimulation and self-direction are positively related to intellect. On a conceptual level, stimulation and self-direction seem more fitting to the intellect dimension, i.e., people might actively seek out situations which are stimulating and call for creative and independent thinking (i.e., search for intellectual stimulation). Behaviors that have been associated with self-direction and stimulation are among others breaking out of the routine to engage in some stimulating task or actively seeking out information to form an opinion about current news-topics. Both behaviors can on a conceptual level be related to situations high in intellect.

A study by Sagiv and Schwartz (2004) found that conservation values were associated with pursuing conventional career paths, while openness-to-change values were associated with pursuing artistic and investigative professions. Additionally, conservation values were negatively associated employees’ beliefs and tendency to act creative at work, while the opposing pattern emerged for openness-to-change values (Dollinger et al., 2007; Kasof et al., 2007).

A sub-facet of the universalism value is broad-mindedness, which contains the belief that others should be free to express their ideas and views (Hunt and Miller, 1968). This idea is also contained in the intellect item “Situation affords an opportunity to express unusual ideas or points of view” (Rauthmann et al., 2014). In addition, the three values are adjunctive values, and as such they should relate to similar outer constructs.

### Adversity and Deception

We assume that power, achievement and stimulation are positively related to the experience of adversity and deception. People who attribute high importance to showing competence, having control over others or seeking stimulation should be more likely to seek out competitive or risky situations, i.e., situations high in adversity. In line with the proposed assumptions of the circumplex value model and the findings mentioned above, conservation as well as self-transcendence should be negatively related to adversity and deception.

### Mating

Mating seems to be more of a basic evolutionary motif and therefore we do not assume that it relates to any specific value.

### Positivity and Negativity

We do not assume that any specific value is related to positivity and negativity. These situation characteristics focus more affective aspects of situations than for example duty. Considering these affective perception, we assume that there are more based on a fit between personal values and opportunities in a situation. That means if a situation fits with an individual’s values than the situation should be perceived as having the potential for a pleasant experience. Contrary, if there is a misfit between personal values and opportunities in a situation than people should perceive the situation as containing more potential for negative feelings (Biber et al., 2008). Additionally, negativity was primarily correlated with neuroticism (Rauthmann et al., 2014) which in turn was only marginally related to any values (Roccas et al., 2002).

### Sociality

Stimulation and self-direction should be positively related to sociality. Regarding the content, sociality is particularly tied to the trait extraversion (Rauthmann et al., 2014), which in turn is consistently associated with openness-to-change values (Roccas et al., 2002; Fischer and Boer, 2015; Parks-Leduc et al., 2015).

In addition, we assume to find roughly the same variability in situation experiences as found in previous studies (i.e., 70%; Sherman et al., 2015). To test our assumptions, we conducted a study using the Day Reconstruction Method (DRM) to examine relations between values and situation characteristics in everyday life.

## Materials and Methods

### Participants and Procedure

The study consists of two samples. The first sample consisted of 154 US-American participants (87 women, *M_age_* = 36.1 years) who were recruited via Amazon Mechanical Turk. Participants received $3 for their participation. The second sample consisted of 84 German undergraduate students (52 women, *M_age_* = 22.9 years) who were recruited at Ulm University and received 2€ (approximately $2.50) as compensation. Overall, we analyzed the data of 238 participants (139 women, *M_age_* = 29.5 years) to investigate the relation between basic human values and situation characteristics in everyday life. Data was collected online using the survey software Unipark. Participants first answered several questionnaires including basic human values and subjective well-being and were then asked to recall their activities and contacts on their last working day. In a last step they answered structured questions about the activities on their last working day. The American sample received English versions of the questionnaires, and the German sample German versions. Data collection was part of a bigger project; therefore, we only report the measures relevant for this article.

### Instruments

#### Basic Human Values

The importance participants attributed to each of the 10 values as guiding principles in their life was measured using the Portrait Values questionnaire (PVQ; Schwartz et al., 2001). In Sample 1 a short version with 21 items (Schwartz et al., 2001) and in Sample 2 a long version with 57 items was used (Schwartz et al., 2012). Each item consists of a description of a person (“portrait”) and respondents rate how similar they see themselves to the portrayed target person on a scale ranging from (*1) very dissimilar* to *(7) very similar* (in Sample 2 the scale ranged from (*1) very dissimilar* to *(6) very similar*). A self-direction sample item reads “Thinking up new ideas and being creative is important to him. He likes to do things in his own original way.” In Sample 1, alpha reliabilities of the PVQ indexes ranged from Cronbach’s alpha = 0.43 (tradition) to 0.77 (stimulation). Considering that the PVQ-21 scale only consisted of two items per scale (3 items for universalism), the internal consistencies are satisfying. In Sample 2, alpha reliabilities of the PVQ indexes ranged from Cronbach’s alpha = 0.57 (hedonism) to 0.87 (benevolence). For the reported statistical analyses, we computed ipsative value scores as recommended by Schwartz (1992). Ipsative scores represent the relative importance of one value compared to the other values instead of the absolute importance.

#### The Day Reconstruction Method

The original DRM-material (Kahneman et al., 2004) consists of three sub-sets; we used the original *Set 2* and a revised form of *Set 1* and *Set 3* in our study. First, participants were presented with the PVQ (i.e., *Set 1*). Then, in *Set 2*, participants were instructed to complete a diary referring to their last working day. Usually the last working day was also the previous day, however, some MTurk workers participated on a Monday, therefore we especially instructed them to think about their last working day. Participants were asked to write down their day by structuring it in chronological episodes. Like in the original DRM instructions, we instructed people to think about their day as if they were watching a movie and so each “movie scene” could be an episode. Participants were told that there is no predefined frame of what constitutes an episode, rather the beginning and end of an episode could be connoted by a change in location, a change in interaction partners or change in activities. After reading the instructions, participants were presented with a maximum of 30 open text items (10 for the morning, 10 for midday, 10 for the evening). It was not possible to enter the notes for the evening episodes before the notes of the morning episode to ensure that participants reported in a chronological order. For each episode, participants indicate the duration and made personal notes. They were informed that the notes were completely private and that the researchers would not read or analyze their personal notes. The notes were only presented to them in *Set 3* to support their recall process. Finally, in *Set 3*, participants answered structured questions about each episode. For each episode they selected what they were doing (14 categories, e.g., commuting) and who they had contact with (7 categories, e.g., spouse), multiple responses were possible. In addition, participants reported their affect during each episode and the situation characteristics of each episode. Finally, participants rated their day as a whole on a scale from (1) *terrible* to (9) *wonderful*. In total, the 238 participants reported 2936 episodes (Sample 1: 1899, Sample 2: 1037). That is on average 12 reports per participant.

#### The 8 DIAMONDS

In Sample 1, situation characteristics were measured using the S-8 (Rauthmann and Sherman, 2016). The S-8 captures the 8 DIAMONDS with one item per dimension. A Duty sample item reads “Does work need to been done?” Participants were asked to rate how characteristic the items were for the situation they had just reported on a 7-point Likert scale ranging from *(1)* *extremely uncharacteristic* to *(7)* *extremely characteristic* *of this situation*. In Sample 2, we used the RSQ-32 inventory (Rauthmann et al., 2014), which includes four items per dimension. Responses were given on a 9-point Likert scale ranging from *(1)* *extremely uncharacteristic* to *(9)* *extremely characteristic of this situation*.

## Results

In both samples and in line with previous findings (Schwartz and Bardi, 2001), benevolence and self-direction were the values attributed with the most importance; tradition and power were the values attributed with the least importance. Additionally, in both samples, mean situation experience was also remarkably similar. Throughout the day, situations high in positivity were most common, followed by situations high in duty and sociality, while experiencing situations high in adversity and deception was relatively rare. These results are in line with previous findings about the 8 DIAMONDS using the ESM (Rauthmann et al., 2014; Sherman et al., 2015), and indicate that people were on average able to reconstruct their memories successfully. Descriptive data is displayed in **Table 1**.

**Table 1:** Mean, standard deviation and variance observed in the 8 DIAMONDS.

	D	I	A	M	O	N	D	S
**Sample 1**								
Mean	3.82	2.67	1.45	2.15	4.87	2.05	1.41	3.69
SD	1.22	1.26	1.04	1.31	1.14	1.27	1.14	1.46
Variance	1.50	1.59	1.07	1.72	1.30	1.62	1.31	2.14
**Sample 2**								
Mean	4.22	3.41	1.88	2.43	4.68	2.84	2.13	3.84
SD	1.19	1.12	1.12	1.33	1.03	1.14	1.11	1.19
Variance	1.41	1.26	1.24	1.76	1.06	1.29	1.23	1.41

*D, duty; I, intellect; A, adversity; M, mating; O, positivity; N, negativity; D, deception; S, sociality. In Sample 1, situation characteristics were measured on a 7-point Likert scale ranging from 1 = completely uncharacteristic to 7 = completely characteristic. In Sample 2, situation characteristics were measured on a 9-point Likert scale ranging from 1 = completely uncharacteristic to 9 = completely characteristic.*

### Preliminary Analysis

To obtain a first understanding of the relation between values and situation characteristics in daily life, we conducted Pearson’s correlations between values and situation characteristics. All correlations are displayed in **Tables 2**, **3**.

**Table 2:** Correlations of the 10 types of values.

	UN	BE	CO	TR	SC	PO	AC	HE	ST
**Sample 1**									
UN	–								
BE	0.17*	–							
CO	-0.23**	0.05	–						
TR	-0.11	-0.18*	0.21**	–					
SC	-0.06	0.20*	0.25**	-0.003	–				
PO	-0.45***	-0.38***	-0.02	-0.23**	-0.28***	–			
AC	-0.46***	-0.23**	-0.13^+^	-0.17*	-0.17*	0.32***	–		
HE	-0.17*	-0.20*	-0.37***	-0.18*	-0.30***	-0.03	0.18*	–	
ST	-0.15^+^	-0.35***	-0.40***	-0.16*	-0.60***	0.22**	0.05	0.30***	–
SD	0.31***	0.10	-0.30***	-0.19*	0.12	-0.28***	-0.35***	-0.17*	0.12
**Sample 2**									
UN	–								
BE	0.20^+^	–							
CO	-0.24*	-0.15	–						
TR	-0.33**	-0.18	-0.06	–					
SC	-0.41***	-0.37**	-0.09	0.22^+^	–				
PO	-0.39***	-0.57***	-0.03	0.40***	0.08	–			
AC	-0.57***	-0.23*	0.01	0.11	0.26*	0.33**	–		
HE	-0.24*	0.21^+^	-0.13	-0.20^+^	-0.09	-0.05	0.05	–	
ST	0.12	0.16	-0.36**	-0.43***	-0.21^+^	-0.13	-0.06	0.19	–
SD	0.30*	0.21^+^	-0.32**	-0.44***	-0.24*	-0.44***	-0.17	-0.13	0.28*

*^+^p < 0.1*, ^∗^*p ≤ 0.05*, ^∗∗^*p < 0.01*, ^∗∗∗^*p < 0.001; UN, universalism; BE, benevolence; CO, conformity; TR, tradition; SC, security; PO, power; AC, achievement; HE, hedonism; ST, stimulation; SD, self-direction.*

**Table 3:** Correlations of the 8 DIAMONDS with the 10 types of values.

	D	I	A	M	O	N	D	S
**Sample 1**								
UN	0.01	-0.09	-0.24**	-0.15^+^	0.07	-0.14^+^	-0.25**	-0.07
BE	-0.12	-0.18*	-0.20*	-0.05	0.09	-0.13^+^	-0.18*	0.15^+^
CO	0.11	0.13	0.15^+^	0.07	-0.12	0.18*	0.17*	0.07
TR	0.12	0.18*	0.16*	0.15^+^	-0.00	0.16*	0.11	0.05
SC	-0.15^+^	-0.15^+^	-0.22**	-0.14^+^	0.09	-0.21**	-0.23**	0.01
PO	0.04	0.12	0.25**	0.15^+^	-0.13	0.15^+^	0.26**	-0.10
AC	-0.02	0.06	0.13	0.13	0.09	0.04	0.16*	0.06
HE	0.02	0.06	0.10	-0.02	-0.00	0.02	0.08	0.07
ST	0.05	0.07	0.16*	0.05	-0.06	0.09	0.13	-0.04
SD	-0.09	-0.25**	-0.33***	-0.23**	-0.00	-0.20*	-0.30**	-0.15^+^
**Sample 2**								
UN	0.04	0.14	-0.02	-0.04	-0.01	0.09	0.03	-0.11
BE	-0.05	-0.09	-0.17	-0.00	0.11	-0.12	-0.22^+^	0.12
CO	0.04	0.10	-0.22	-0.24	0.09	-0.00	-0.06	-0.01
TR	0.20^+^	0.11	0.26*	0.20^+^	-0.12	0.23*	0.26*	0.20^+^
SC	0.06	-0.03	0.09	0.09	-0.08	0.09	0.04	0.02
PO	-0.04	0.02	0.30*	0.16	-0.14	0.07	0.23*	-0.01
AC	-0.14	-0.15	-0.05	-0.02	0.03	-0.14	-0.11	-0.00
HE	-0.09	-0.21^+^	-0.03	0.07	0.10	-0.06	-0.01	0.16
ST	-0.04	-0.06	-0.04	0.03	0.01	-0.13	-0.05	-0.09
SD	0.14	0.10	-0.07	-0.02	0.22^+^	-0.11	-0.13	-0.02

*^+^p < 0.1*, ^∗^*p ≤ 0.05*, ^∗∗^*p < 0.01*, ^∗∗∗^*p < 0.001; UN, universalism; BE, benevolence; CO, conformity; TR, tradition; SC, security; PO, power; AC, achievement; HE, hedonism; ST, stimulation; SD, self-direction; D, duty; I, intellect; A, adversity; M, mating; O, positivity; N, negativity; D, deception; S, sociality.*

#### Relations Between Values

Overall, in both samples the relations are in line with the model’s assumptions, that is adjunctive values are positively related while opposing values are negatively correlated with each other. However, there were some unusual relations. In Sample 1, tradition was not significantly related to security. Also self-direction was negatively related to hedonism and not significantly related to stimulation in Sample 1. In Sample 2, conformity was negatively related to opposing values (i.e., self-direction and stimulation), however, it was not significantly related to adjunctive values (i.e., tradition and security).

#### Relations Between Values and Situation Characteristics

In Sample 1, universalism as well as benevolence were significantly negatively related to adversity, negativity and deception. Universalism was also marginally significant negatively related to mating, while benevolence was negatively related to intellect and marginally significant positively to sociality. Conformity and tradition were positively related to adversity and negativity. Conformity was also positively related to deception, while tradition was negatively related to intellect and marginally significant positively with mating. Security showed the opposite pattern, that is, it was negatively related to all situation characteristics, expect for positivity and sociality (none significant relations). Power was positively related to adversity, negativity and deception. Achievement was only positively related to deception. No significant relation between hedonism and any of the 8 DIAMONDS emerged. Stimulation was only positively related to adversity. Self-direction was negatively related to all DIAMONDS, except for duty and positivity (none significant relations). In Sample 2, only a few significant associations emerged. Benevolence was negatively related to deception. Conformity was negatively related adversity and mating. Tradition was positively related to all DIAMONDS, except for intellect and positivity (none significant relations). Power was significantly positively related to deception. Hedonism was significantly negatively related to intellect. No significant relations emerged for universalism, security, achievement, stimulation and self-direction. In sum, the pattern differs immensely between the samples. Possible explanations and implications are discussed in the general discussion.

### Main Analysis

In both samples episodes were nested within participants, therefore all following analyses used multilevel modeling with participants as nested factors. First, we estimated unconditional cell mean models for each situation characteristic to analyze how much variability in the experience of situation characteristics was between versus within participants. The variance components, intraclass correlations (ICC), intercepts and number of observation for each analysis are displayed in **Table 4**. All situation characteristics displayed sizeable between person variance (Sample 1: τ_00_, *M* = 1.24, *SD* = 0.26; Sample 2: τ_00_, *M* = 1.04, *SD* = 0.18), but even larger within person variance (Sample 1: σ, *M* = 2.58, *SD* = 1.9, Sample 2 σ, *M* = 2.83, *SD* = 1.46). The ICCs ranged from 0.14 to 0.85 (*M =* 0.42) in Sample 1 and from 0.14 to 0.51 in Sample 2 (*M* = 0.31). Compared to studies using the ESM (Sherman et al., 2015), the resulting ICCs for adversity, negativity and deception differed greatly indicating that for those situation characteristics differences in experience were mainly explained by individual differences instead of differences between the reported episode. For all other situation characteristics most variance was due to differences between episodes.

**Table 4:** Variance components, intraclass correlations (ICC), intercepts and number of observation.

Scales	Variance between intercepts (between-person variance)	Variance around intercepts (within-person variance)	ICC (proportion of variance between persons divided by total variance)	Intercept = fixed effects intercept from unconditional cell means model	*n* = number of reported episodes
**Situation characteristics**					
**Sample 1**					
Duty	0.92	5.53	0.14	3.80	1899
Intellect	1.19	3.12	0.28	2.64	1899
Adversity	1.03	0.41	0.71	1.45	1899
Mating	1.36	3.20	0.30	2.15	1899
Positivity	0.99	2.35	0.30	4.88	1899
Negativity	1.44	1.25	0.53	2.04	1899
Deception	1.27	0.23	0.85	1.41	1899
Sociality	1.69	4.56	0.27	3.70	1899
**Sample 2**					
Duty	1.00	4.09	0.20	4.08	1037
Intellect	0.98	3.50	0.22	3.37	1037
Adversity	1.01	0.99	0.51	1.86	1037
Mating	1.46	2.26	0.39	2.40	1037
Positivity	0.93	2.43	0.28	4.64	1037
Negativity	1.08	2.56	0.30	2.83	1037
Deception	1.04	1.35	0.44	2.11	1037
Sociality	0.89	5.43	0.14	3.84	1037

*N_*Sample 1*_ = 154, N_*Sample 2*_ = 84*.

Next, we used values as predictors of situation experience by estimating “means-as-outcomes” regression models (Cohen et al., 2003). That means, we predicted each DIAMONDS score with the value hypothesized to be associated with. Our analytic approach is based on the analyses by Sherman et al. (2015). The results for each model are displayed in **Table 5** (Sample 1) and **Table 6** (Sample 2). The indices of fit for the models are also reported in the **Tables 5**, **6**. The marginal R (R_m_) can be interpreted as the model fit for only the fixed effects, while the conditional R (R_c_) can be interpreted as the overall fit of the model (Nakagawa and Schielzeth, 2013). We give one detailed example, i.e., predicting the experience of intellect from the value benevolence in Sample 1. The fixed average experienced intercept for intellect was 2.65 with a standard deviation of 1.08, indicating that although the experienced intellect was on average rather low, there were large individual differences in the amount of intellect experienced with a slope of -0.20, which was statistically significant (*p* ≤ 0.05). This means for every one-point increase in the importance attributed to benevolence, we would expect a 0.20 decrease in the average level of experienced intellect.

**Table 5:** Means-as-outcomes regression models in Sample 1.

Situation characteristic	b	LL	UL	t	R_m_	R_c_
Duty	3.80	3.61	3.99		0.05	0.38
Conformity	0.12	-0.04	0.27	1.14		
SD in intercepts	0.95	0.78	1.14			
SD in residuals	2.35	2.28	2.43			
Duty	3.80	3.60	3.98		0.05	0.38
Tradition	0.12	-0.03	0.26	1.46		
SD in intercepts	0.96	0.79	1.12			
SD in residuals	2.35	2.28	2.42			
Duty	3.80	3.62	4.02		0.06	0.38
Security	-0.13	-0.31	0.04	-1.52		
SD in intercepts	0.96	0.78	1.13			
SD in residuals	2.35	2.27	2.42			
Duty	3.80	3.61	3.99		0.02	0.38
Achievement	-0.04	-0.22	0.13	-0.41		
SD in intercepts	0.96	0.79	1.12			
SD in residuals	2.35	2.28	2.42			
Intellect	2.64	2.46	2.85		0.05	0.53
Universalism	-0.10	-0.30	0.11	-1.04		
SD in intercepts	1.09	0.94	1.24			
SD in residuals	1.77	1.71	1.82			
Intellect	2.65	2.45	2.81		0.09	0.53
Benevolence	-0.20	-0.40	-0.01	-2.06*		
SD in intercepts	1.08	0.93	1.22			
SD in residuals	1.77	1.70	1.82			
Intellect	2.64	2.45	2.84		0.08	0.53
Conformity	0.14	-0.03	0.31	1.66^+^		
SD in intercepts	1.08	0.94	1.24			
SD in residuals	1.77	1.70	1.82			
Intellect	2.64	2.45	2.82		0.10	0.53
Tradition	0.18	0.02	0.33	2.16*		
SD in intercepts	1.08	0.92	1.21			
SD in residuals	1.77	1.71	1.82			
Intellect	2.64	2.43	2.87		0.04	0.53
Stimulation	0.05	-0.08	0.19	0.74		
SD in intercepts	1.09	0.93	1.24			
SD in residuals	1.76	1.70	1.82			
Intellect	2.65	2.45	2.84		0.14	0.53
Self-direction	-0.31	-0.48	-0.10	-3.22**		
SD in intercepts	1.06	0.89	1.19			
SD in residuals	1.77	1.71	1.82			
Adversity	2.53	1.97	3.08		0.20	0.85
Universalism	-0.17	-0.34	-0.01	-2.06*		
Age	-0.03	-0.04	-0.01	-3.13**		
SD in intercepts	0.98	0.84	1.08			
SD in residuals	0.64	0.62	0.66			
Adversity	2.65	2.01	3.21		0.17	0.85
Benevolence	-0.17	-0.31	-0.01	-2.14*		
Age	-0.03	-0.04	-0.01	-3.59***		
SD in intercepts	0.96	0.84	1.07			
SD in residuals	0.64	0.62	0.66			
Adversity	2.61	2.01	3.19		0.20	0.85
Conformity	0.13	-0.02	0.28	1.95*		
Age	-0.03	-0.05	-0.02	-3.93***		
SD in intercepts	0.96	0.84	1.07			
SD in residuals	0.64	0.62	0.67			
Adversity	2.70	2.10	3.30		0.20	0.85
Tradition	0.16	0.02	0.29	2.41**		
Age	-0.03	-0.05	-0.02	-4.11***		
SD in intercepts	0.96	0.86	1.07			
SD in residuals	0.64	0.62	0.67			
Adversity	2.45	1.90	3.08		0.19	0.84
Security	-0.12	-0.27	-0.01	-1.73^+^		
Age	-0.03	-0.04	-0.01	-3.12**		
SD in intercepts	0.96	0.86	1.07			
SD in residuals	0.64	0.62	0.66			
Adversity	2.60	2.03	3.15		0.28	0.84
Self-direction	-0.29	-0.44	-0.14	-3.56***		
Age	-0.02	-0.04	-0.01	2.99**		
SD in intercepts	0.93	0.82	1.04			
SD in residuals	0.64	0.62	0.66			
Adversity	2.59	2.12	3.21		0.21	0.85
Power	0.15	0.03	0.25	2.45*		
Age	-0.03	-0.04	-0.01	-3.32**		
SD in intercepts	0.95	0.84	1.05			
SD in residuals	0.64	0.62	0.66			
Deception	2.59	1.89	3.28		0.23	0.92
Universalism	-0.21	-0.38	-0.03	-2.23*		
Age	-0.03	-0.05	-0.01	-3.08**		
SD in intercepts	1.06	0.93	1.18			
SD in residuals	0.48	0.46	0.50			
Deception	1.40	1.25	1.59		0.16	0.92
Benevolence	-0.20	-0.36	-0.01	-2.21*		
SD in intercepts	1.11	0.99	1.25			
SD in residuals	0.48	0.46	0.50			
Deception	2.69	2.07	3.35		0.16	0.92
Conformity	0.18	0.02	0.31	2.34*		
Age	-0.03	-0.05	-0.02	-3.94**		
SD in intercepts	1.06	0.94	1.19			
SD in residuals	0.48	0.46	0.50			
Deception	1.40	1.23	1.58		0.21	0.92
Security	-0.22	-0.39	-0.06	-2.91*		
SD in intercepts	1.10	0.98	1.23			
SD in residuals	0.48	0.46	0.50			
Deception	2.67	2.07	3.27		0.28	0.92
Self-direction	-0.28	-0.47	-0.10	-3.12**		
Age	-0.03	-0.04	-0.01	-3.06**		
SD in intercepts	1.05	0.93	1.16			
SD in residuals	0.48	0.46	0.50			
Deception	2.66	2.04	3.32		0.15	0.92
Power	0.18	0.05	0.31	2.72**		
Age	-0.03	-0.05	-0.01	-3.26**		
SD in intercepts	1.06	0.93	1.17			
SD in residuals	0.48	0.46	0.50			
Deception	1.41	1.24	1.59		0.15	0.92
Achievement	0.16	0.01	0.33	2.03*		
SD in intercepts	1.12	0.98	1.23			
SD in residuals	0.48	0.46	0.50			
Sociality	3.70	3.48	3.92		0.02	0.52
Stimulation	-0.03	-0.21	0.13	-0.42		
SD in intercepts	1.30	1.10	1.49			
SD in residuals	2.14	2.06	2.21			
Sociality	3.70	3.47	3.94		0.09	0.52
Self-direction	-0-0.23	-0.47	-0.03	-1.97*		
SD in intercepts	1.28	1.1	1.48			
SD in residuals	2.14	2.07	2.21			

*N_*Sample 1*_ = 154. bs are unstandardized multilevel regression coefficients. Ipsative value scores were used. Situation characteristics were measured on a 1–7 scale. LL and UL represent lower and upper limits for 95% confidence intervals, respectively. R_*M*_, marginal R; R_*C*_, conditional R; ^+^p < 0.1, ^∗^p < 0.05, ^∗∗^p < 0.01, ^∗∗∗^p < 0.001.N_*Sample 2*_ = 75. bs are unstandardized multilevel regression coefficients. Ipsative value scores were used. Situation characteristics were measured on a 1–9 scale. LL and UL represent lower and upper limits for 95% confidence intervals, respectively. R_*M*_, marginal R; R_*C*_, conditional R; ^+^p < 0.1, ^∗^p < 0.05, ^∗∗^p < 0.01, ^∗∗∗^p < 0.001.*

**Table 6:** Means-as-outcomes regression models in Sample 2.

Situation characteristic	b	LL	UL	t	R_m_	R_c_
Duty	4.13	3.85	4.37		0.01	0.46

Conformity	0.03	-0.36	0.44	0.16		
SD in intercepts	1.03	0.80	1.25			

SD in residuals	2.00	1.91	2.08			
Duty	4.13	3.87	4.37		0.08	0.46
Tradition	0.18	-0.06	0.42			

SD in intercepts	1.01	0.77	1.23			

SD in residuals	2.00	1.91	2.09			
Duty	4.13	3.86	4.44		0.04	0.46
Security	0.14	-0.25	0.54	0.65		
SD in intercepts SD in intercepts	1.02	0.78	1.24			
SD in residuals	2.00	1.91	2.09			

Duty	4.13	3.88	4.39		0.09	0.46

Achievement	-0.24	-0.54	0.07	-1.59		
SD in intercepts	1.00	0.77	1.21			
SD in residuals	2.00	1.91	2.09			

Intellect	3.39	3.14	3.61		0.09	0.47
Universalism	0.35	-0.12	0.76	1.54		

SD in intercepts	0.97	0.76	1.16			

SD in residuals	1.87	1.78	1.96			
Intellect	3.39	3.13	3.63		0.06	0.47

Benevolence	-0.22	-0.66	0.29	-0.94		
SD in intercepts	0.98	0.76	1.18			
SD in residuals	1.87	1.78	1.94			

Intellect	3.39	3.12	3.65		0.06	0.47
Conformity	0.18	-0.21	0.56	0.95		
SD in intercepts	0.98	0.76	1.20			
SD in residuals	1.87	1.78	1.95			
Intellect	3.39	3.11	3.64		0.04	0.47
Tradition	0.08	-0.17	0.33	0.63		
SD in intercepts	0.99	0.78	1.19			
SD in residuals	1.87	1.79	1.94			
Intellect	3.39	3.12	3.64		0.03	0.47
Stimulation	-0.07	-0.36	0.20	-0.50		
SD in intercepts	0.99	0.78	1.23			
SD in residuals	1.87	1.79	1.94			
Intellect	3.39	3.14	3.64		0.06	0.47
Self-direction	0.21	-0.22	0.61	1.01		
SD in intercepts	0.98	0.77	1.19			
SD in residuals	1.87	1.78	1.95			
Adversity	1.89	1.63	2.12		0.02	0.72
Universalism	-0.04	-0.43	0.34	-0.19		
SD in intercepts	1.05	0.86	1.23			
SD in residuals	0.99	0.95	1.04			
Adversity	2.3	1.77	2.83		0.15	0.72
Benevolence	-0.39	-0.85	0.04	-1.8^+^		
SD in intercepts	1.02	0.85	1.2			
SD in residuals	0.99	0.95	1.04			
Adversity	1.89	1.65	2.14		0.13	0.72
Conformity	-0.29	-0.65	0.05	-1.59		
SD in intercepts	1.03	0.86	1.20			
SD in residuals	0.99	0.95	1.04			
Adversity	2.16	1.81	2.48		0.19	0.72
Tradition	0.27	0.07	0.49	2.40*		
SD in intercepts	1.00	0.83	1.17			
SD in residuals	0.99	0.95	1.04			
Adversity	1.89	1.65	2.13		0.09	0.72
Security	0.19	-0.19	0.53	0.99		
SD in intercepts	1.04	0.86	1.24			
SD in residuals	0.99	0.95	1.04			
Adversity	1.89	1.67	2.13		0.07	0.72
Self-direction	-0.17	-0.55	0.21	-0.85		
SD in intercepts	1.04	0.84	1.23			
SD in residuals	0.99	0.95	1.04			
Adversity	2.43	1.97	2.96		0.21	0.72
Power	0.35	0.04	0.61	2.50*		
SD in intercepts	1.00	0.83	1.19			
SD in residuals	0.99	0.95	1.04			
Deception	2.11	1.85	2.34		0.03	0.68
Universalism	0.09	-0.31	0.51	0.40		
SD in intercepts	1.04	0.86	1.23			
SD in residuals	1.13	1.08	1.19			
Deception	2.54	1.97	3.01		0.15	0.68
Benevolence	-0.42	-0.86	0.05	-1.91^+^		
SD in intercepts	1.02	0.82	1.19			
SD in residuals	1.13	1.08	1.18			
Deception	2.11	1.88	2.37		0.05	0.68
Conformity	-0.12	-0.47	0.27	-0.62		
SD in intercepts	1.04	0.84	1.22			
SD in residuals	1.13	1.08	1.19			
Deception	2.34	1.98	2.65		0.15	0.68
Tradition	0.23	0.01	0.43	2.00**		
SD in intercepts	1.02	0.83	1.2			
SD in residuals	1.13	1.08	1.18			
Deception	2.11	1.86	2.35		0.04	0.68
Security	0.09	-0.29	0.52	0.48		
SD in intercepts	1.04	0.87	1.23			
SD in residuals	1.13	1.07	1.18			
Deception	2.11	1.88	2.36		0.07	0.68
Self-direction	-0.17	-0.57	0.20	-0.84		
SD in intercepts	1.04	0.86	1.21			
SD in residuals	1.13	1.08	1.18			
Deception	2.53	2.04	3.05		0.15	0.68
Power	0.27	-0.03	0.56	1.9^+^		
SD in intercepts	1.02	0.84	1.23			
SD in residuals	1.13	1.08	1.18			
Deception	2.11	1.86	2.37		0.08	0.68
Achievement	-0.14	-0.42	0.13	-1.01		
SD in intercepts	1.04	0.84	1.20			
SD in residuals	1.13	1.08	1.19			
Sociality	3.82	3.56	4.08		0.04	0.38
Stimulation	-0.11	-0.40	0.18	-0.75		
SD in intercepts	0.95	0.69	1.15			
SD in residuals	2.32	2.21	2.44			
Sociality	3.83	3.54	4.08		0.02	0.38
Self-direction	0.06	-0.35	0.46	0.28		
SD in intercepts	0.95	0.70	1.17			
SD in residuals	2.32	2.22	2.43			

*N_Sample 2_ = 75. bs are unstandardized multilevel regression coefficients. Ipsative value scores were used. Situation characteristics were measured on a 1–9 scale. LL and UL represent lower and upper limits for 95% confidence intervals, respectively. R_M_, marginal R; R_C_, conditional R; ^+^p < 0.1, ∗p < 0.05, ∗∗p < 0.01, ∗∗∗p < 0.001.*

#### Sample 1

Benevolence and self-direction predicted a significant decrease in experienced intellect, while conformity and tradition predicted a significant increase in experienced intellect. Power, conformity, tradition and stimulation predicted a significant increase in experienced adversity, while benevolence, universalism, security and self-direction predicted a significant decrease in experienced adversity. Achievement and conformity predicted a significant increase in experienced deception, while self-transcendence, security, and self-direction predicted a significant decrease in experienced deception. Benevolence predicted a significant increase in experienced sociality, while self-direction predicted a significant decrease in experienced sociality. Controlling for age and gender, we found that gender was a significant predictor of duty, i.e., women experienced more duty. Age predicted a significant decrease in adversity and deception.

#### Sample 2

Power and tradition predicted a significant increase in experienced adversity. Self-direction predicted a significant increase in experienced positivity. Tradition predicted a significant increase in experienced deception. There was no influence of age or gender.

The results concerning relation between values and intellect reveal a reversed pattern than hypothesized. The results for adversity and deception are at least partly in line with our assumptions. While the relation between the self-enhancement-self-transcendence dimension was clear and mostly as expected, the relation between the openness-to-change-conservation dimension was more inconclusive. Namely, not all values belonging to same higher dimension showed the same relation, which is contrary to the assumed compatibilities in the circumplex model. Overall the pattern of results suggests that in both samples individual differences in values are at least to some extent associated with differences in situation experiences in everyday life. However, unfortunately the results did not replicate and therefore, no clear pattern emerged. Possible reasons and implications for these findings are further discussed in the general discussion section.

## Discussion

The investigation of human values and their relation to behavior has been an on-going topic in psychology (Roccas and Sagiv, 2010). Values are supposed to serve as guidelines in peoples’ life (Schwartz, 1992), and thus it seems naturally that they should strongly relate to peoples’ behavior. However, up to date, the link between values and actual behavior, i.e., not self-reported behavior, is weak or even non-existed (Fischer, 2017). There have been several attempts to explain this missing link. For example, some researchers assumed that in order for values to influence behavior they need to be activated (Maio, 2010; Sagiv et al., 2011). Others researchers have argued that values are too abstract to actually determine one single behavior or even that behavior cannot actually be assigned to a specific value because there might be different understandings of which behavior actually represents a value depending on social or cultural backgrounds (i.e., value instantiations; Hanel et al., 2017). Goal of the present work was to contribute to the value-behavior link discussion by providing a novel approach, i.e., measuring subjective situation experiences, i.e., the situational 8 DIAMONDS, to better understand situational factors that may influence the value-behavior link. Even so, we did not investigate any kind of behavior, we will first discuss the present results, the limitation of the studies and then there potential meaning for the value-behavior link.

First, we reported the relations between values and subjective situation experiences. Overall, the pattern of correlations between samples was quite different. We found many relations in Sample 1, unfortunately there were only few relations in Sample 2. While self-transcendence values were negatively related to all negative situation characteristics (i.e., deception, adversity and negativity) in Sample 1, in Sample 2 only benevolence was negatively related to deception. Power was in both samples related to adversity and deception, but there emerged no clear pattern for achievement. In Sample 1, security was strongly negatively related to almost all situation characteristics, while tradition and conformity only showed moderate relations and in the opposite direction. Interestingly, the conflicting value self-direction also was negatively related to almost all situation characteristics. Due to the circumplex model, we assumed that opposing values would show opposite relations with the same characteristic resulting in a sinusoid curve (Schwartz, 1992). However, the results might indicate that maybe conflicting values shift or shape peoples’ perception in the same way. As a consequence, this similar perception might result in different pattern of emotional and behavioral outcomes. For example, both valuing security and self-direction was associated with lower experience of situations high in intellect. Experiencing that a situation is low in intellect might active an individual high in self-direction to leave the situation or evoke negative feelings and emotions. Contrary, experiencing that a situation is low in intellect might active an individual high in security to stay in the situation or evoke positive feelings. However, the conflicting values benevolence and power did show opposing relations with the same situation characteristics. Therefore, the results provide neither strong evidence for the typical sinusoid curve nor for the idea that opposing values might shift perception in a similar way.

Interestingly, duty, positivity and sociality did not show any strong relations with values. One possible explanation could be that situation characteristics captured with the 8 DIAMONDS differ in their objectivity. The results by Rauthmann et al. (2014) showed that adversity and deception had the lowest interrater reliability. This could indicate that some DIAMONDS leave more room for interpretation that is subjective experience due to individual differences than others. In an ambiguous situation individual differences might influence the perception of potential threats more than the perception of having a task to attend to. However, in that case it would be surprising that positivity is not related to values as it also relates to more subjective experience. Other measurements have been developed and future research should examine if using the other instruments, which capture situation characteristics with only adjective might be better suited (overview: Horstmann et al., 2017). Overall, the correlations pattern differed immensely and should be treated with caution.

Considering the results concerning the ICCs, they show that individual differences especially influence the experience of negatively connoted situation experiences, i.e., most variance in the experienced adversity, deception and negativity was due to individual factors and not due to specific situational aspects. This could indicate that values do indeed transcend specific situation in daily life and are a *lens* through which people see and interpret their surroundings. In Sample 1, our results show that benevolence predicts less aversive and deceptive situation experience in daily life, while the opposing pattern emerged for power as a predictor. Unfortunately, this pattern could not be replicated in Sample 2.

From a psychological perspective, the relation between subjective situation experiences and values might be more interesting than the relation to actual activities or contacts. The findings suggest that values are not necessarily used to evaluate a specific action or situation; rather they may refer to a proneness to see situations in certain way. If this is the case our findings could be used to predict how people with different values will experience identical situations, i.e., situations which are standardized. For example, to investigate cooperative behavior researchers often rely on decision-making in economic games like the prisoner’s dilemma or the trust game (e.g., Camerer, 2011). The games do have objective differences (e.g., number of players, information certainty), however, if values do shape the perception of situations, including those standardized scenarios, we would expect that subjective experiences of different games are more similar for one individual compared to the experiences of another. For example, an individual valuing power might be prone to experience most economic games as deceptive situations compared to people valuing benevolence. The differences in situation experience may also serve as a mediator between values and behavior.

Although, considering prior research (Sherman et al., 2015) the variance due to individual differences in our samples was much higher. One possible explanation could be methodical differences between DRM and ESM. While ESM uses momentary assessment to capture brief events, the DRM uses a memory technique to recall all the events on a typical day. Even so, due to the specific technique recall biases and memory distortions are reduced, they cannot be completely excluded. Some studies show that in general negative events are easier to recall (Porter et al., 2010), and that the recall is also associated with personality traits (Martin et al., 1983). Moreover, using ESM can lead to overestimated brief events and distortions due to sample bias (Kahneman et al., 2004). Think about the situation teaching a class, using ESM participants might never report this episode as they will probably not stop teaching in order to fill out a questionnaire. Using DRM participants will probably report this episode as part of the day. These methodical differences provide some explanation for the differences between our findings and previous findings (Sherman et al., 2015). Both methods have their strengths and depending on the research question one or the other might be more useful.

Another point worth of discussing, is that, values belonging to the same higher dimension did not always relate to situation experience in similar manner. Although, that might seem surprising, one should keep in mind that even if values are compatible and belong to the same higher dimension, they do represent distinct motivational goals. Power and achievement are both self-enhancement values, but only power is related to experiencing adversity, i.e., threats and conflict. In general, people assume that others have a similar motivation than themselves (Ockenfels and Raub, 2010). Thus, one explanation could be that people who value power often (unconsciously) assume that others want to challenge their dominate role, which in turn leads to a perceived threat.

Furthermore, some values which are supposed to be compatible (i.e., security, tradition, and conformity) showed relations to the 8 DIAMONDS in the opposite direction. The findings contradict the assumption of the circumplex value model. Situation selection in everyday life could be an explanation for the contradictive results. Especially, security often showed a different pattern than conformity and tradition. Security refers to valuing the status quo and a safe surrounding; therefore, it seems plausible that people valuing security experienced less negative and aversive situations. It is opposed to their underlying motivational goal to put themselves in situations which might entail threats. On the other hand, valuing conformity and tradition implies being obedient to socially imposed expectations. Thus, people may find themselves in situations which are unpleasant, however, due to social expectations they stay in the situation. Past studies have already shown that individual differences (i.e., personality traits and personal values) are linked to the exposure of objective life events (Magnus et al., 1993; Paunonen, 2003; Sortheix et al., 2013). Therefore, it seems to be more likely that individual differences might also represent a proneness to experience certain situations characteristics but not determining them.

Contrary to our hypothesis, no value was correlated or predicted the experience of duty. Moreover, additional analyses revealed that in Sample 1 gender, but not age, predicted experienced duty, that is women reported more situations high in duty than men. Considering the sample, it could be that with certain life events (e.g., full-time working, having children) more situations high in duty become part of a daily routine. Another explanation could be that all of our participants reported a week day, which might be determined by situations or tasks which cannot be actively chosen. Maybe value relations to duty, but also to the other situation characteristics may be enhanced or even be opposed to our findings during the weekend, i.e., during times in which people can actively shape their day. Opposed to our assumptions, we further found that self-direction was negatively and tradition positively related to experienced intellect. Again, we believe that the pattern might change during the weekend. People valuing self-direction may not experience intellect during daily routine, while people valuing tradition may even experience daily routine as stimulating and intellectual challenging. Our assumptions were mainly based on the theoretical idea that people are consciously or unconsciously seeking out situations which fit their values. Research in the work context supports this idea, showing that values influence amongst others career choices (Sagiv and Schwartz, 2004). However, we did not find that for example people valuing stimulation also experience more stimulating situations. It seems worthwhile to investigate the relation between values and situation selection over a couple of days in future research.

### Limitations

As mentioned above, participants may consciously or unconsciously seek out different situations, e.g., situations which enable them to fulfill their goals or act in accordance with their goals. Situation selection (Rauthmann et al., 2014) implies that people actually experience different situations. Unfortunately, our data does not allow drawing any conclusions about active situation selection. Participants only reported which situations they encounter, but we do not know if they actually put themselves into the situation. Furthermore, our data does not allow drawing any conclusion about how people actually perceive the identical situation, i.e., a standardized situation in which the same cues are present. One major limitation is that we cannot draw any conclusions about situation selection and situation construal in daily life.

Furthermore, we have no behavioral data in daily life. However, we believe that our data provides some initial evidence and can inspire future research. For example, one could easily extend the DRM to capture self-reported behavior but also it would be possible to add some items to ask about active situation selection. In addition, comparing *in situ* and *ex situ* ratings of the situation descriptions given in the DRM could provide some clue about the relation between values and situation construal.

However, considering the recent problems concerning the replicability of psychological findings the major limitation is that we could not replicate our findings in the second sample. We chose our samples for theoretical and practical reasons (i.e., availability of a student sample). On a theoretical level to investigate how values relate to situation experiences in daily life, it seemed useful to have samples which differed in several aspects (e.g., nationality, profession, and age) to potentially obtain more generalizable findings. One reason could be that not only did the samples differ in their demographics, but also we used different instruments to measure values and situation characteristics in both samples. Maybe, the results would have been more similar if the studies had not differed on all three aspects. We chose our samples for theoretical and practical reasons (i.e., availability of a student sample). On a theoretical level to investigate how values relate to situation experiences in daily life, it seemed useful to have samples which differed in several aspects (e.g., nationality, profession, and age). Even so, if a real effect exists and the instruments are valid, the differences in results between the samples should not have been so pronounced. Furthermore, both samples are quite small, which probably entails a low power, and thus in order to find an effect it would need to be large effect. Given the very broad conceptualization of both values and situation characteristics, it seems more realistic to assume a small effect. Additionally, we conducted multiple testing which – without corrections – might lead to an inflation of the alpha error. Thus, the present results should be taken with caution and be seen as some initial evidence that points in the direction of values being related to subjective situation experience. A lot of further research is needed to make any strong or reliable statements.

### Implications for the Value-Behavior Link

Previous research has shown that individual differences in situation perception also transfer to differences in behavior (Rauthmann et al., 2014). However, as we have no real behavioral data in our study, we cannot affirm this assumption for our data. In the future to better understand and maybe to bridge the value-behavior gap, it might be worth to examine the relation between value consistent behavior and situation selection. Situation selection could have similar effects as value activation on value consistent behavior. People who consciously or unconsciously put themselves in competitive situations might activate self-enhancement values. At the same time self-enhancement values might become more important because people want to appear consistent and therefore infer from their behavior to their values (Fischer, 2017).

Moreover, the novel taxonomies to measure situation perception can also be used to examine the relations between values and behavior in a standardized given situation, that is in an objective identical situation. There are several possibilities through which in an identical situation experienced situation characteristic might mediate the relation between values and behavior. For example, differences in behavior might emerge due to differences in the experience of the same characteristics. In a social dilemma situation, the subjective experience of deception might influence the willingness to behave on a prosocial manner. However, it is also possible that people behave in the same way due to different situation experiences. In a social dilemma, some people might act prosocial because they experience low adversity and are therefore not afraid to be exploited. Others might act prosocial because they experience high duty, and thus they feel it is their task to contribute. Motivation, which includes values, relates to decisions (conscious or unconscious) that involve how, when, and why people engage in behavior (Pinder, 1998). Overall, we believe that focusing more on subjective situation experiences due to values, could provide novel understandings of when and why allocate effort to a task or activity.

## Conclusion

Since 2014, five different instruments to capture situation characteristics have been published (Horstmann et al., 2017). This development shows, that currently subjective situation experiences is a continuously developing field and provides novel insight to understand peoples’ behavior. We believe that it is worth to examine and understand the precise aspects in situations which may activate or prevent value-consistent behavior.

In conclusion, we believe that situation characteristics are a useful tool to understand and measure external factors that influence the value-behavior link. Our work provides some initial evidence that behavior is a function of situation and person, and thus that in order to close the gap between values and behavior, a better understanding of this interaction is necessary. Therefore, to understand why people act or do not act in accordance with their values, we first need to obtain a better understanding of the situation they experience.

## Ethics Statement

The study was conducted in full accordance with the Ethical Guidelines of the German Association of Psychologists (DGPs) and the American Psychological Association (APA). No personal information was assessed; participants remained completely and were not identified in any regard during the study process. Moreover, by the time the data were acquired in July 2016, it was also not customary at Ulm University, nor at most other German universities, to seek ethics approval for simple, non-invasive field studies.

## Author Contributions

RK and JK conceived of the presented idea, and carried out the experiment. RK performed the computations. JK verified the analytical methods. RK wrote the manuscript with support from JK.

## Conflict of Interest Statement

The authors declare that the research was conducted in the absence of any commercial or financial relationships that could be construed as a potential conflict of interest.
